# Solar-powered Scholarship

**DOI:** 10.1007/s42438-022-00372-7

**Published:** 2022-12-02

**Authors:** Sharon Boyd

**Affiliations:** The Royal (Dick) School of Veterinary Studies, The University of Edinburgh, Edinburgh, UK

**Keywords:** Researcher’s fiction, Academic fiction, Science fiction, Sci-Fi, Educational futures, Education fiction, Social science fiction

## Dara, Sitting Under a Beech Tree by a River in Scotland

The wind rustles through the leaves, the sound of water gently flowing past. In the distance, a blackbird begins to sing. Not much of a dawn chorus at this time of year, but there was rain overnight, and that guarantees happy blackbirds. With her back to the beech tree, Dara watches as the riverbank breathes in the sunlight. Grey shadows turn to green moss and rippled bark, the first sparkling bubbles of a trout rising. She gets up and stretches; it is harvest time and it is every hand to the task, so it will be a busy day. Coming here early is her peaceful think-time, allowing her senses time to wake with the new day.

A skein of geese threads across the sky, coming to spend winter. She has three meetings today and it is too dark under this tree to pick up enough sunlight to run her kit reliably in the morning. Leaving the river behind, she walks back to the house. Factoring in time zones, she has scheduled meetings with Embla and Alani this morning, and back to the tree in the evening when the batteries are fully charged to call Chan Bai. She has got data stories to submit to the research hub, so that means heading up to the beacon this evening. There is still enough light to work with the Natalie digital heliograph. A few weeks from now, the team will be breaking out the signal lamps as the nights get darker. Just a couple of transfers from here to the main University campus, and she is keen to see who else might be gathering at the beacon tonight.

Home now, with the sounds of the town waking up. Welig has left the solar kettle out for her, so she gathers some leaves from the pots for her teacup and pours the hot water. As the steam rises, she boots up her system, idly watching the traffic passing along the street. Not much car or bus traffic anymore, mostly bikes, horses, and carts. Logging in, she can see Embla on standby waiting for their meeting. They have got the sun before Dara has today.

## Embla, Cooking Dinner in a Small House, Northernmost Tip of Norway

It was good to touch base with Dara today, she is a good academic mentor. It is been a long journey to the placement, but Embla has finally been able to back up their travel log. Not many people travel far these days, since the pandemics, so they had to accept work along the way and take advantage of the opportunities that came up to reach the final destination. Dara reminded Embla that the journey is part of the learning process, they adapt to different voices, scents, and sights as they move through the landscape.

Embla spent yesterday with the Sámi grandmothers; they said something the same. As a listener-in-training, Embla has been finding it hard to accept that they cannot capture everything. That is why it was important to come here. The grandmothers teach a different way of listening, not listening to capture but to learn with, become with, and connect to. Sing the bird, not of the bird. Embla is excited to see what this new understanding will bring to their research, what they can share with the learning cohort, and how this will contribute to the listening research on the global open research network.

Tomorrow they will be going on a long hike to introduce Embla to the land, so they will all need a good hearty meal tonight. Embla brings knowledge from their family group to share in turn, reciprocity and respect. Embla’s mother-kin skills in electronics and mechanical engineering to fix some of the kit that the local team were finding troublesome and unreliable; father-kin plant-growing wisdom with seeds that may do well in the new northern climate. And this dumpling stew recipe, is ready now for the oven, to slow cook all day. Embla plans to add the dumplings later. They have brought herbs from their father’s garden to flavour them, gifting a taste of their home in the south. Embla smiles – everything is south of here, only the sea is north. Their fiddle is in their pack, so they will tune that and bring it along tonight too.

Dara said she was going to talk to Alani today – that reminds Embla to pin her a message to let her know they are thinking of her. Like Embla, Alani is in a new place; unlike Embla, she did not choose to travel, to leave her home. As Embla wraps the dumplings and puts them in the cold store, they hope Alani is OK.

## Alani, Standing on the Borderland Between the Farm and the Prairie, Midwestern North America

Alani stands looking out over the prairie after her meeting with Dara, taking a pause before going in to help with breakfast. Her home is under the ocean, and the people in this landlocked state have given her refuge. The hurricanes damaged homes on the islands first, cut power and communication, impacted transport and water supplies. Initially, there was support from other nations, but then came the time where everyone was too focused on keeping their own people and countries secure. Other pandemics followed Covid-19, so travel was limited, and communication was fractured. The open network maintained by the universities became the lifeline, a reliable way to reach others on the mainland as well as around the world. The heartbreak days came where the people knew there was nothing that could be done, not enough land remaining to support the island residents. The boats took all they could carry; seed banks and storage of all kinds were transported in an effort to preserve land, species, culture, and memory.

And so she is standing on the edge of a prairie, watching the grasses and plants move in the breeze and remembering the sound of the ocean, imagining salt scents on the breeze. It is the storms that remind her most of home. When the tornadoes come, the thunder of air and rain and wildness is the nearest thing to the hurricanes that marked the last days on her islands. Her islands; the islands that made her.

That is her role now – to speak for the islands. Arranged through her studies at the University, and with Dara’s help, she is working with Ed of the Chocktaw nation and Sue from the Osage nation. Ed holds the space for her to talk of her grief. Or not talk. To sometimes sit together watching the prairie waves in good company, listening to the not-an-ocean and what it has to tell her.

Sue is more practical. Her way of listening-learning is to walk the land, letting each plant speak its message of healing. She keeps a running commentary, usually speaking more to the plants than to Alani. She gets Alani moving on days she would rather hide, and time spent on her feet with her nose to the earth has brought a different kind of healing. Both Sue and Ed are part of the community networks that restored the prairies, linking up tiny remaining pockets and patching the gaps from seedbanks, stories, and hard work.

Alani’s studies have changed over the years she has been in the University. Where once she listened to the voices of her own land to preserve them, now she listens to others talk of restoration. These lessons may help her people one day in the future to bring lives back to her islands. For now, this place and these people have welcomed her. Ed reminded her the other day that the islands live through her; she is and speaks the islands that made her. She breathes the prairie air deep, closes her eyes, and listens. The sounds remind her of an old story her grandmother told her of storms and rainbows. She smiles and makes a note in her journal; she will bring that to the next community meeting to share and remember.

## Chan Bai, Sitting by a Small Fire in the Early Hours of the Morning in a Tropical Rainforest, Vietnam

It is an early start today – or a late one, he thinks wryly, depending on your viewpoint. Chan Bai has been working with the local human, snail, and crab communities. Plus the occasional elephant, but mostly the smaller creatures.

Dara is up late too, meeting with Chan Bai in one of his breaks from night listening after her research meeting at the beacon. She is full of chat about the latest research that had come out from the central group and local stories from those that gathered on the hill tonight to upload their data stories and songs to the network. She also talks about the harvest and the change of pace at this time of year when every other task is set aside while her community prioritises gathering the foods that will feed them for the winter and be traded with other towns. Everything is part of the work of a listener, so no doubt she will have other tales when the harvest is done.

Chan Bai feels less lonely listening to her. Not that he was alone here – he has friends and family nearby, including the species he loves working with. But sometimes it is lonely at night on the walk through the jungle. Quiet but not quiet, with the many voices of bug, bird, reptile, and amphibian, like being alone in a crowd of people who do not want to talk to you.

He tells her about his research, his way of listening to species that humans do not yet know how to speak to. Over time, research has shown the many ways of hearing, and he is aware of his limited human listening abilities. For now, his work looks at listening to other species in the community that rely on – or avoid – the crabs and snails, including the tales from his local community and the others that are a day’s walk away. His human community rely on the snails and crabs as one of their harvests, and he is the link between the species to ensure human need is balanced with the survival of the species he respects and cares for, and the habitats on which they all depend.

He speaks of the slow work of rainforest restoration that seems to be going well, though his parents still talk of the species that were lost before the global switch. The song of the gibbons is missing from the morning, they say, and he wonders aloud if the jungle misses the song. Dara asks if he will be out much longer tonight – it is twilight where she is and she is winding down after a long day. It is a few hours yet before dawn comes, and he will stay a little longer. He will rest tomorrow and do some work in the village before he packs for his biannual short trips to visit listeners in nearby villages. They pick a date for their next meeting when he is back home and her town has a break in harvesting.

Both he and Dara pause, each listening to the voices of other species carried by microphones so many miles apart. Dara listens to the song of the rainforest at night, the hum of many voices is soothing. Chan Bai imagines the river she has spoken of as the Scottish night falls. The wind rustles through the leaves, the sound of water gently flowing past. In the distance, a blackbird begins to sing.

